# Is There Evidence Supporting the use of Dermatoglyphics as an Auxiliary Diagnostic Method for Periodontitis? A Scoping Review

**DOI:** 10.1155/ijod/5546489

**Published:** 2026-10-08

**Authors:** Sara Maria do Amaral Melo, Maria Teresa Andrade do Amaral, Maria Luiza Lima Santana, Túlio Eduardo Nogueira, Virgílio Moreira Roriz

**Affiliations:** ^1^ Postgraduate Program in Dentistry Federal University of Goiás Goiânia Goiás Brazil ufg.br; ^2^ Faculty of Dentistry and Postgraduate Program in Dentistry Federal University of Goiás Goiânia Goiás Brazil ufg.br

**Keywords:** dermatoglyphics, diagnosis, fingerprint patterns, periodontitis

## Abstract

**Aims:**

To identify and synthesize the scientific evidence regarding the use of dermatoglyphics as an auxiliary method in the diagnosis of periodontitis.

**Methods:**

This scoping review was conducted in accordance with the Joanna Briggs Institute (JBI) guidelines and reported following the PRISMA Extension for Scoping Reviews (PRISMA‐ScR) checklist. Multiple electronic databases and sources of gray literature were searched without language or time restrictions. Studies evaluating individuals with periodontitis using dermatoglyphics as a complementary diagnostic approach were included. Study selection, data extraction, and analysis were independently performed by two reviewers. The risk of bias was assessed using the JBI critical appraisal tools for analytical cross‐sectional studies, with disagreements resolved by a third reviewer.

**Results:**

A total of 24 studies were included, comprising 21 cross‐sectional studies and three case–control studies. Considerable heterogeneity was observed in both periodontal diagnostic criteria and fingerprint collection methods. None of the included studies reported diagnostic accuracy measures for dermatoglyphic patterns (DPs) in comparison with a clinical periodontal diagnosis. A predominance of the ulnar loop fingerprint pattern was frequently reported among individuals with periodontitis, although some studies also described a higher prevalence of whorl patterns.

**Conclusions:**

Although the results may suggest an association between certain DPs and the presence of periodontitis, the current evidence is insufficient to support its clinical application. There is a clear need for well‐designed multicenter studies with methodological rigor and standardized diagnostic criteria.

## 1. Introduction

Dermatoglyphics is a term coined by Cummins and Midlo in 1926 to define the study of ridge patterns of the skin on the fingers, toes, palms, and soles [1, 2]. Dermatoglyphic patterns (DPs) are defined as the configurations of the skin on the digits, palms, toes, and soles. This so‐called volar skin is covered by thin parallel ridges (papillary lines) that enhance grip and assist in texture discrimination [3–5]. The overall ridge pattern is unique to each individual, not duplicated in any other person, including monozygotic twins, or even across different sites of the same individual, due to differential growth and intrauterine pressure [6, 7].

DPs can be classified into three major categories. The arch, the simplest pattern, occurs in approximately 5% of cases. In this form, ridges flow from one side to the other without forming curves. The loop pattern is the most common and is found in about 70% of fingerprints. It is characterized by a triradius, with the ridge pattern entering and exiting on the same side, and at least one recurved ridge between the core and the triradius. The whorl pattern is found in roughly 25% of cases and consists of two or more triradii, where the ridges form at least one complete circuit [1, 3, 6].

DPs have traditionally been used for individual identification because of their characteristics of uniqueness, permanence, social acceptability, ease of use, and low cost, among others [8, 9]. These same features also define ideal diagnostic tests. Studies have shown strong associations between DPs and the identification of syndromic and genetically inherited diseases such as leukemia, diabetes, hypertension, and epilepsy since genetic and chromosomal alterations during intrauterine development affect ridge formation [10–12].

More recently, DPs have been investigated in various fields of dentistry, considering that oral tissues and skin ridges share a common origin in the ectodermal layer. As both are strongly, though not exclusively, genetically determined, it has been hypothesized that the same factors influencing alterations in oral structures may also produce distinctive dermatoglyphic features [13–16]. Studies suggest that whorl and loop patterns may reflect genetic susceptibility and predisposition to dental caries [14], while loop and arch patterns appear more frequent among individuals with oral squamous cell carcinoma [17]. In addition, Class II craniofacial patterns have been associated with an increased frequency of arches and ulnar loops, along with a reduced frequency of whorls. Conversely, Class III malocclusions tend to show increased arches and radial loops with reduced ulnar loops [15].

Periodontitis is a chronic multifactorial inflammatory disease associated with the presence of biofilm and characterized by progressive destruction of the tooth‐supporting structures. Despite being associated with the presence of microbiological factors, systemic diseases, environmental, and immunological factors, genetic alterations also play an important role in the etiopathogenesis of periodontal diseases, recent diagnostic methods to identify the genetic basis of periodontitis are costly and require high technical rigor in their execution [18–20].

Some studies suggest that dermatoglyphics may serve as a potential auxiliary diagnostic method for periodontal diseases [21, 22]. However, well‐designed studies investigating the relationship between dermatoglyphics and periodontal diseases remain scarce and inconclusive, highlighting the importance of synthesizing the available evidence and identifying existing gaps. Therefore, this review aimed to identify and synthesize the scientific evidence on the use of dermatoglyphics as an auxiliary method in the diagnosis of periodontitis.

## 2. Methods

### 2.1. Protocol and Registration

This study was conducted in accordance with the Joanna Briggs Institute (JBI) Manual for Evidence Synthesis [23], and the report was prepared following the PRISMA Extension for Scoping Reviews (PRISMA‐ScR) checklist (Supporting Information 1: File 1). The study protocol was registered on the Open Science Framework (OSF) platform (https://doi.org/10.17605/OSF.IO/5CV3Q).

The PCC mnemonic (population, concept, and context) was applied to formulate the research question of this scoping review, as follows: (P) population: patients with periodontitis; (C) concept: use of dermatoglyphics as an auxiliary method for the diagnosis of periodontitis; and (C) context: the literature, with no language, geographic, or temporal restrictions. Therefore, the research question guiding this scoping review was as follows: “what evidence is available on the use of dermatoglyphics as an auxiliary method for the detection of periodontitis?”

### 2.2. Eligibility Criteria

Studies were included if they reported at least one group of patients with periodontitis and considered dermatoglyphics as a diagnostic method for periodontitis. The following were excluded: book chapters, reviews, letters to the editor, conference abstracts, case reports, in vitro or animal studies, opinion papers, narrative literature reviews, and case series with four or fewer cases, as proposed by the classification of Abu‐Zidanet al. [24].

### 2.3. Information Sources and Search Strategy

Individual searches were conducted up to October 03, 2025. The search strategy was applied to the following databases: MEDLINE (PubMed), EMBASE, Scopus, Web of Science, Cochrane Library, LILACS, and LIVIVO. Additionally, gray literature was searched in ProQuest Dissertations & Theses Global and Google Scholar. For Google Scholar, the first 100 results ranked by relevance were considered [25]. No restrictions were applied regarding language, year of publication, or publication status.

Supplementary strategies were also considered, such as the creation of database alerts (alerts were monitored until May 25, 2025–October 31, 2025 and manual searches in journals considered particularly relevant to the field of interest: Journal of Clinical Periodontology, Journal of Periodontology, and Journal of Periodontal Research. Furthermore, the reference lists of all included publications were examined to identify additional potentially eligible studies.

The search strategy was developed in consultation with a librarian experienced in dental research. It was initially designed for the PubMed database and subsequently adapted for each of the other databases (Supporting Information 2: File 2). Combinations of controlled vocabulary (MeSH, EMTREE, and DeCS) and free‐text keywords were used. For LILACS and Google Scholar, in addition to English terms, Portuguese and Spanish vocabulary were also considered in the construction of the search strategy.

After the search, all records were exported to the online software Rayyan (Qatar Computing Research Institute: https://www.rayyan.ai/), where duplicate records were removed, followed by the screening and selection of abstracts and full texts.

### 2.4. Study Selection, Data Extraction, and Synthesis

Two authors (Sara Maria do Amaral Melo and Maria Teresa Andrade do Amaral) independently screened the titles and abstracts of the retrieved records. Studies meeting the eligibility criteria were assessed in full to confirm inclusion. Any disagreements were resolved through consensus with a third author (Maria Luiza Lima Santana).

For data extraction, the following information was collected from each included publication: author, year of publication, study design, study objective, sample size, participant characteristics (age and sex distribution), dermatoglyphic assessment method, periodontal diagnostic criteria adopted, and main findings. In addition, data related to diagnostic accuracy were extracted whenever available, including measures such as sensitivity and specificity. Data extraction was conducted by two authors (Sara Maria do Amaral Melo and Maria Teresa Andrade do Amaral), and the results were verified by a third author (Maria Luiza Lima Santana).

When full‐text articles were unavailable or data were incomplete, corresponding authors were contacted via e‐mail on three attempts. The synthesis of the included studies was carried out through narrative description and presented in tables and figures.

### 2.5. Risk of Bias Assessment

Although the risk of bias assessment is not mandatory in scoping reviews, we opted to assess it to describe the methodological quality of the included studies. This additional step aimed to provide complementary information regarding the robustness of the available evidence.

The methodological quality of the included studies was independently assessed by two reviewers (Sara Maria do Amaral Melo and Maria Teresa Andrade do Amaral). The JBI Critical Appraisal Checklist for Analytical Cross‐Sectional Studies [26] was used for cross‐sectional studies, and the JBI Critical Appraisal Checklist for Case‐Control Studies was applied to case–control studies [26]. Disagreements in the assessment were resolved through discussion until consensus was reached, or, when necessary, by consultation with a third reviewer (Maria Luiza Lima Santana).

The JBI checklist for cross‐sectional studies comprises 8 items: (1) clarity in criteria for inclusion, (2) description of study subjects and setting, (3) validity and reliability of exposure measurement, (4) use of objective and standardized criteria for condition measurement, (5) identification of confounding factors, (6) strategies to manage confounding factors, (7) validity and reliability in measuring outcomes, and (8) appropriateness of statistical analysis. The JBI checklist for case–control studies comprises 10 items distributed across the same domains, with the addition of the domains of temporal precedence [27].

The items were rated as “yes,” “no,” or “unclear” in accordance with JBI guidelines. “Yes” indicated that the methodological criterion was adequately met and that the corresponding source of bias did not substantially influence the study findings; “no” indicated that the criterion was not met, suggesting an increased risk of bias in that methodological domain; and “unclear” was assigned when there was insufficient information to allow for a reliable assessment. Thus, the evaluation focused on the extent to which each study sought to minimize potential sources of bias related to participant selection, exposure and outcome measurement, confounding factors, and statistical analysis, providing a domain‐structured assessment of the internal validity of the included studies [27, 28].

The overall risk of bias classification was determined using criteria predefined by the authors: studies with all items rated as green or with up to one yellow domain were considered high quality (low risk of bias); studies with two yellow domains or up to one red domain were considered moderate quality (moderate risk of bias); and studies with two or more red domains were considered low quality (high risk of bias) [28].

To graphically display the results of the risk of bias assessment, the tool robvis [29] (https://www.riskofbias.info/) was used, generating a traffic light plot. The plot presents, for each study and checklist item, the risk of bias classification coded by colors: green (low risk of bias), yellow (unclear risk of bias), and red (high risk of bias).

## 3. Results

### 3.1. Evidence Source Selection

A detailed overview of the study selection process is presented in Figure 1. The electronic search strategy retrieved 840 publications, of which 394 remained after duplicate removal and were screened by the reviewers. At the full‐text assessment stage, 27 publications were assessed for eligibility, resulting in the exclusion of four publications. The main reasons for exclusion were the absence of a specific group of participants with periodontal disease and the use of dermatoglyphics as an inclusion criterion rather than as an auxiliary diagnostic method.

**Figure 1:**
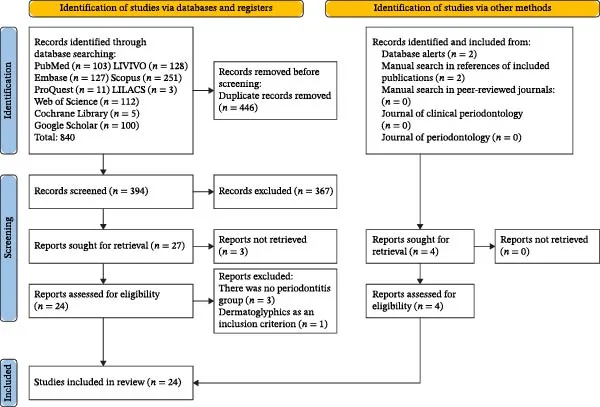
Flow diagram for study selection.

Despite three unsuccessful attempts to contact the authors, the full texts of three abstracts could not be obtained, and these were therefore excluded [30–32]. Additionally, search strategies, manual searches of reference lists and journals, and database alerts led to the inclusion of four further studies. In total, 24 studies were included in this scoping review.

### 3.2. Evidence Characteristics

Of the 24 publications included in this review, three were case–control studies [33–35] and 21 were cross‐sectional studies [18, 20, 21, 36–53]. The included studies were published between 2005 and 2024, with nine (37.5%) being published from 2020 onward.

Twenty‐two (91.7%) studies originated from a single country, India, with one study conducted in Turkey [36] and one study in Brazil [53]. In total, 3586 individuals were included across these studies, with sample sizes ranging from 34 [47] to 800 participants [18]. The mean age of participants was reported in only seven studies [18, 20, 38, 49, 50, 52, 53], ranging from 27 to 53.7 years. Notably, 72.7% of the studies did not report participant age data.

Regarding sex distribution, data were available in 10 studies (36.4%). Across these, 50.4% (*n* = 982) of participants were male and 49.5% (*n* = 965) were female [18, 20, 36, 41, 44, 46, 50, 52, 53] and Khara et al. [49] reported a male‐to‐female ratio of 4:13.

Periodontal diagnosis of the included studies was determined based on the following parameters: gingival index [18, 48, 51], plaque index [36, 44, 48], bleeding on probing [18, 44, 46, 48, 49, 52], probing depth [18, 20, 21, 34, 36, 40, 41, 44, 46, 49, 51–53], clinical attachment loss [20, 21, 34–36, 39, 40, 44, 46, 49, 51–53], Russell’s periodontal index [20, 40, 47, 51], number of teeth with bone loss [20, 21, 36], simplified oral hygiene index [41, 51], Greene and Vermillion oral hygiene index [18], community periodontal treatment needs index [42], and community periodontal index [35]. Six studies did not report which periodontal parameters were considered to classify the periodontal status of participants [33, 37, 38, 43, 45, 50].

The most frequently used method for fingerprint collection was the ink‐and‐paper technique (17 studies; 70.8%) [18, 20, 21, 33–39, 42–44, 48, 49, 51, 52]. Five studies employed digital biometric scanners [40, 46, 47, 50, 53]; in one study, participants’ fingerprint patterns were directly examined by the researchers using a magnifying lens [45]; and in one study, the collection method was not reported [41].

None of the included studies reported diagnostic accuracy measures for DPs in comparison with a clinical periodontal diagnosis. Although several studies suggested a potential predictive or screening role for dermatoglyphics, their analyses were limited to measures of association and group comparisons without a formal evaluation of diagnostic test performance.

The detailed characteristics of the included studies are presented individually in Table 1.

**Table 1:** Characteristics and main results of the included studies.

Author/year	Study design	Study location	Main objective	Sample	Periodontal parameters	Dermatoglyphic method used	Results
Atasu et al. 2005 [36]	Cross‐sectional	Marmara and Istanbul Universities, Turkey	Not reported	158 participants in four groups: JP (*n* = 36), RPP (*n* = 45), AP (*n* = 38), healthy (*n* = 39)	Specific diagnoses for each type of periodontitis	Ink and Kleen paper; Cummins & Midlo method	JP showed lower frequencies of radial loops (3.0%) and higher prevalence of ulnar loops (52.8%). AP and controls exhibited a similar pattern, with predominant ulnar loops (53.5% and 50.5%) and whorls (36.2% and 41.6%), respectively. No statistically significant differences were observed.
Devishree and Gujjari 2015 [33]	Case‐control	JSS Dental College, Mysore, India	To compare palmar dermatoglyphic features between healthy individuals and patients with aggressive periodontitis	30 participants (15 healthy, 15 with aggressive periodontitis)	Clinical diagnosis of aggressive periodontitis	Black ink on prepared paper; AFIS system	57% ulnar loops and 30% whorls in the aggressive periodontitis group. Similar results were observed in the control group, with ulnar loops (40%) and whorls (36%). Statistical analysis was not reported.
Shyamala et al. 2015 [37]	Cross‐sectional	Government Dental College, Bangalore, India	To identify specific digital patterns in aggressive periodontitis	60 participants (30 with aggressive periodontitis, 30 healthy), aged 20–35 years	Not specified	Black Kajal applied with Eyetex Kajal stick	In the aggressive periodontitis group, increased frequency of double loop whorls on thumbs (60%) and single loop whorls on left ring fingers (60%). Statistical analysis was not reported.
Deotale et al. 2016 [38]	Cross‐sectional	Kalmegh Smruti Dental College, Nagpur, India	To assess dermatoglyphics as a diagnostic tool for generalized chronic periodontitis	120 participants (60 with chronic periodontitis, 60 healthy)	Clinical and radiographic diagnosis; criteria not specified	Blue ink on thick white paper	Ulnar loops in 80.5% of cases with periodontitis; statistically significant difference (*p* < 0.001).
Vaidya et al. 2017 [21]	Cross‐sectional	Dept. of Periodontics and Implantology, Maharashtra, India	To relate fingerprints with chronic periodontitis	200 participants (100 with chronic periodontitis, 100 healthy), 98 men, 102 women	Clinical criteria, bone loss, probing depth	Duplication ink on bond paper	Controls showed a higher prevalence of ulnar loops. In chronic periodontitis, ulnar loops decreased (52.4%) and whorls increased (41.1%), with a higher frequency of whorls on the right hand (*p* = 0.04) and left hand (*p* < 0.05).
Chatterjee et al. 2017 [18]	Cross‐sectional	Periodontics Outpatient Department (OPD), India	To compare fingerprint patterns in patients with generalized chronic periodontitis and generalized chronic gingivitis	800 participants (437 with chronic periodontitis, 363 with chronic gingivitis)	Gingival index (Löe & Silness), oral hygiene index (Greene & Vermillion), probing depth	Blue ink pad method	The most common pattern in the periodontitis group was ulnar loop (*n* = 164/37.53%), followed by central pocket whorl (*n* = 158/36.16%). Statistical analysis was not reported
Elavarasu et al. 2017 [39]	Cross‐sectional	Periodontics OPD, JKKN Dental College & Hospital, Komarapalayam, India	To analyze palmar dermatoglyphic patterns in patients with chronic periodontitis	80 participants (40 healthy, 40 with chronic periodontitis)	Clinical attachment loss used to define periodontitis cases	Standard ink method, using blue duplication ink, thick white paper, and sponge	Loops were the most prevalent pattern in the periodontitis group (104; 26.0%), followed by whorls (89; 22.25%). The whorl pattern was approximately twice as frequent compared with the healthy group (41; 10.25%). No statistical analysis was reported.
Astekar et al. 2017 [40]	Cross‐sectional	Institute of Dental Sciences, Bareilly, Uttar Pradesh, India	To compare dermatoglyphic patterns between controls and periodontitis patients	60 participants (30 with chronic periodontitis, 30 healthy)	Clinical attachment loss >5 mm and PD >3 mm in >30% of sites	Biometric scanner and flatbed scanner	The control group showed a higher frequency of loops (59.0% vs. 48.0%; *p* = 0.007). Conversely, whorls were more common in the periodontitis group (48.34% vs. 36.67%; *p* = 0.004).
Kranti et al. 2018 [41]	Cross‐sectional	Ramaiah University of Applied Sciences, Bangalore, India	To correlate fingerprint patterns with periodontal status	60 participants (30 healthy, 30 with chronic periodontitis)	Clinical criteria with PD ≥ 5 mm	Not detailed; Swedish classification method	Ulnar loops were the most prevalent dermatoglyphic pattern in both groups and on both hands. No statistical correlation analysis was performed between dermatoglyphic patterns and periodontal status.
Mukherjje et al. 2018 [42]	Cross‐sectional	Nair Hospital Dental College, Mumbai, India	To evaluate cheiloscopy and dermatoglyphics as predictors of caries and periodontitis	200 participants in four groups (70 with caries, 58 with periodontal disease, 14 with both, and 58 healthy)	DMFT, CPITN, DMFT index	Ink pad; Galton–Henry classification	Significant association between digital patterns and oral diseases. Loop pattern on the left thumb most prevalent (71.4%) in participants with caries and mild‐to‐moderate periodontal disease, statistically significant compared to other groups.
Mukherjje et al. 2018 [43]	Cross‐sectional	Dept. of Periodontics, India	To use dermatoglyphics as a risk indicator for diabetes in patients with periodontitis	75 individuals (25 healthy, 25 with periodontitis, 25 with periodontitis + diabetes mellitus)	Not specified	Ink; 10‐finger impressions	The whorl pattern was the most prevalent in the periodontitis group (39.60%), followed by the loop pattern (33.20%). Similar results were observed in the control group, with 47.20% whorls and 44.44% loops. The chi‐square test for interdependence was 17.07, with a *p*‐value of 0.001.
Rathod et al. 2018 [44]	Cross‐sectional	VSPM Dental College and Research Centre, India	To compare dermatoglyphic characteristics in healthy and periodontitis groups	45 participants in three groups (healthy, aggressive periodontitis, chronic periodontitis)	Clinical criteria: PD ≥ 5 mm and CAL ≥ 5 mm	Standard ink method, roller impressions	Whorls were more frequent in individuals with periodontitis (43%), in both chronic and aggressive forms, whereas ulnar loops were predominant in healthy individuals (55%). No statistically significant difference was observed.
Grace and Sankari 2019 [45]	Cross‐sectional	Saveetha Dental College, Chennai, India	To correlate dermatoglyphic patterns with periodontal health	45 participants (three groups: healthy, aggressive periodontitis, chronic periodontitis)	Not detailed	Magnifying lens, left hand	Increased whorls in chronic periodontitis and loops in aggressive periodontitis compared to healthy patients. No statistically significant differences overall (*p* = 0.897).
Sai Karthikeyan and Shiva Manjunath 2019 [46]	Cross‐sectional	Periodontics and Implantology OPD, India	To relate fingerprint pattern types with ridge count	60 participants divided into two groups (30 with chronic periodontitis, 30 healthy)	Clinical criteria for chronic periodontitis	Digital scanner for all 10 fingers	Radial loop was the most prevalent pattern in both groups (healthy: 46%; periodontitis: 43%), with no statistically significant difference. Plain whorl (21% vs. 15%) and plain arch (13% vs. 3%) were more frequent in the periodontitis group, both showing statistically significant differences (*p* < 0.05).
Kumar et al. 2019 [47]	Cross‐sectional	Institute of Dental Sciences, Bareilly, Uttar Pradesh, India	To compare fingerprint patterns in chronic periodontitis and healthy individuals	34 participants (17 with periodontitis, 17 healthy)	Russell’s Periodontal Index (scores 6 and 8)	Biometric scanner; Sir Richard Henry classification	The loop pattern was more frequent in the control group (51.18%) than in the periodontitis group (32.94%), with *p* = 0.012. The whorl pattern predominated in the periodontitis group (57.65% vs. 33.53% in controls), with statistical significance (*p* = 0.001).
Paul et al. 2020 [34]	Case‐control	Annor Dental College and Hospital, Kerala, India	To determine correlation between dermatoglyphics and periodontitis	286 participants (143 with periodontitis, 143 healthy)	Probing depth >5 mm, attachment loss >3 mm	Ink on disposable strips, 10 fingers	The loop pattern was the most prevalent in the chronic periodontitis group, showing a statistically significant association between the distribution of dermatoglyphic patterns and the study groups (*p* < 0.05) across the different fingers analyzed.
Abrol et al. 2020 [48]	Cross‐sectional	Periodontics OPD, India	To assess fingerprint patterns and palatal rugae in relation to periodontal disease	120 participants (60 men, 60 women)	Four groups: healthy, gingivitis, chronic periodontitis, aggressive periodontitis	Ink and roller method; Sir Richard Henry classification	Significant increase in the whorl pattern among periodontitis group (55.8%), whereas in control group loop pattern (66.6%) was found to be significantly increased (*p* < 0.05)
Khara et al. 2020 [49]	Cross‐sectional (Pilot study)	Central India	To assess the association between ABO blood group, dermatoglyphics, and periodontal status	102 participants (three groups: healthy, gingivitis, periodontitis)	Standard clinical criteria, including attachment loss	Ink pad, thumbs only	Significant association between left thumbprints and periodontal status.
Ojha et al. 2021 [35]	Case‐control	People’s College of Dental Sciences & Research, Bhopal, India	To explore association between dermatoglyphic patterns and periodontitis	100 participants (70 with periodontitis, 30 healthy)	CPI and attachment loss	Blue ink on thick paper (Strong AM23)	Among periodontitis and control group, there was significant increase in the whorl pattern among periodontitis group (55.8%), whereas in control group loop pattern (66.6%) was found to be significantly increased (*p* < 0.05). Among periodontitis and control group significant difference noted for right hand and left hand palm ‘adt’ (*p* < 0.05)
Kumar et al. 2023 [20]	Cross‐sectional	Rajas Dental College and Hospital, Tamil Nadu, India	To detect variations in fingerprint patterns between localized and generalized chronic periodontitis	300 participants divided into three groups of 100	Russell’s Periodontal Index and clinical criteria	Blue ink and thick white paper	Group I, whorl and loop patterns showed a similar distribution (4.950 ± 3.10 and 4.750 ± 3.09, respectively). Group II demonstrated a prevalence of whorl patterns (5.300 ± 3.37), Group III showed a significantly increased prevalence of loop patterns (5.800 ± 2.72). Statistically significant difference was observed
Patnaik et al. 2024 [50]	Cross‐sectional	Dept. of Periodontics and Implantology, Vishnu Dental College, India	To observe and classify fingerprint patterns and ridges in individuals with different periodontal conditions	114 participants in three groups (38 each: healthy, chronic periodontitis, aggressive periodontitis)	1999 classification, no periodontal parameters detailed	SecuGen WBF Hamster Pro 20 digital scanner; Sir Richard Henry classification	Radial loops are more frequent in chronic periodontitis (28%); plain whorls (26.3%) in aggressive periodontitis. Statistically significant difference was observed (*p* < 0.05).
Imthiyazuddin et al. 2024 [51]	Cross‐sectional	SIBAR Institute of Dental Sciences, Guntur, India	To determine relation between dermatoglyphics and different stages of periodontitis	80 participants in four groups of 20 (Stages I–IV)	Clinical criteria including PD and periodontal indices	10‐finger collection, method not detailed	Ulnar loops prevalent across all stages. Right hand and left hand showed nonsignificant differences with a *p*‐values of 0.18 and 0.24. Statistically significant difference between hands.
Ashifa et al. 2025 [52]	Cross‐sectional	Department of Periodontics, Government Dental College, Tamil Nadu, India	To explore associations among fingerprints, lip prints, blood groups and periodontitis	300 participants (150 healthy; 150 with Stage III, Grade B periodontitis)	Probing depth, clinical attachment loss, bleeding on probing, vertical bone loss	Fingerprints of 10 fingers collected with ink pad; Karu & Jain classification.	The periodontitis group showed higher frequency of whorl patterns in thumbs, index, and ring fingers, while the healthy group showed higher frequency of ulnar loop across all fingers. Statistically significant difference was observed (*p* < 0.05).
Veras et al. 2026 [53]	Cross‐sectional	Dental clinics, Federal University of Goiás, Brazil	To evaluate whether computerized dermatoglyphics (Salus method) can identify fingerprint patterns associated with periodontitis	157 participants (95 healthy; 62 with Stage III–IV periodontitis)	Clinical attachment loss, probing depth	Digital fingerprint scanning of 10 fingers (Watson Mini) analyzed via Salus method.	Ulnar loop was the most prevalent pattern in both groups (30%), followed by whorl, with no statistically significant differences.

Abbreviations: AP, adult periodontitis; CPI, community periodontal index; CPITN, community periodontal index of treatment needs; DMFT, decayed, missing, and filled teeth index; JP, juvenile periodontitis; PD, probing depth; RPP, rapidly progressive periodontitis.

Two cross‐sectional studies [36, 53] were judged to have an overall moderate risk of bias, whereas all other studies cross‐sectional and all studies case–control were classified as having an overall high risk of bias. The risk‐of‐bias assessment for the cross‐sectional studies is presented in Figure 2 and that for the case–control studies in Figure 3.

**Figure 2:**
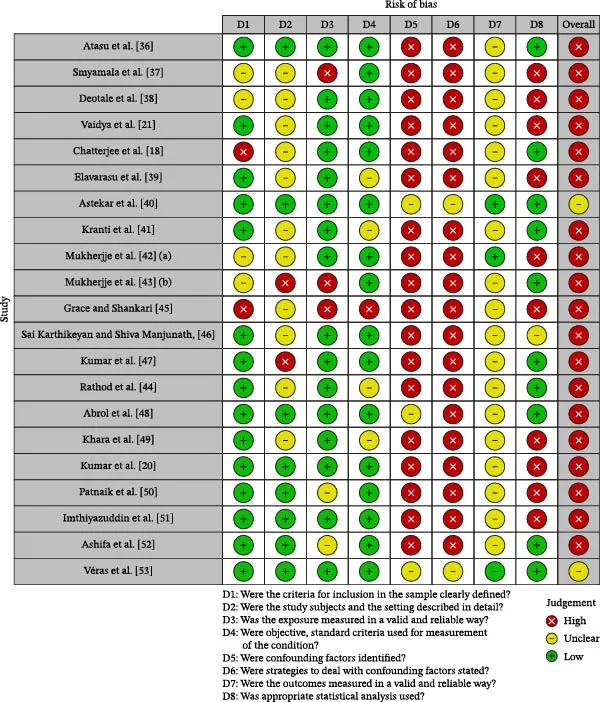
Table of risk of bias of included cross‐sectional studies.

**Figure 3:**
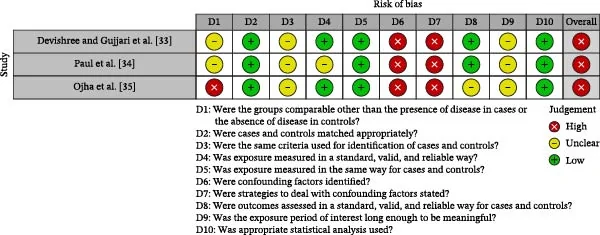
Table of risk of bias of included case–control studies.

The general analysis of the cross‐sectional studies evidenced heterogeneity in the methodological quality of the included studies (Figure 4). The domains with the best performance were those related to the definition of inclusion criteria (15/21 of the included studies –62.5%), exposure measurement (16/21 of the included studies –76.2%), and the use of objective and standardized criteria for outcome measurement (16/21 of the included studies –76.2%), which predominantly presented a low risk of bias. On the other hand, a high risk of bias was observed in the domains concerning the identification (18/21 included studies –85.7%) and treatment of confounding factors (19/21 included studies –90.5%), followed by the domain related to the adequacy of statistical analyses (10/21 included studies –47.6%) [20, 21, 37–39, 42, 45, 49–51].

**Figure 4:**
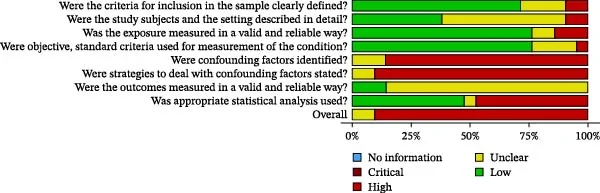
Quality assessment of cross‐sectional studies included.

The case–control studies analyzed presented low methodological quality, with a predominance of high risk of bias in the domains related to the identification and control of confounding factors (3/3 included studies –100%). The domains related to adequate matching between cases and controls (3/3 included studies –100%) and the standardization of exposure measures (D4‐2/3 included studies –66.7% and D5‐ [3/3 included studies –100%]) showed satisfactory performance, with most responses classified as low risk of bias. Finally, the adequacy of statistical analyses (3/3 included studies –100%) was also consistently evaluated as low risk of bias (Figure 5).

**Figure 5:**
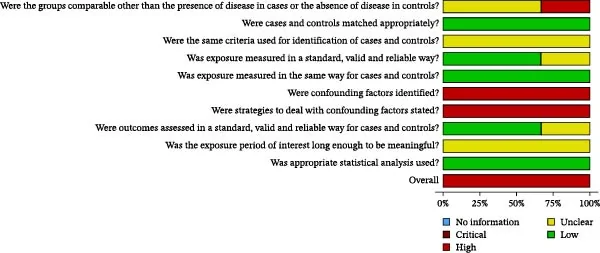
Quality assessment of case–control studies included.

## 4. Discussion

In 2018, the American Academy of Periodontology and the European Federation of Periodontology developed a new classification of periodontal diseases to address the limitations of the Armitage [54] system, which included overlaps and insufficient distinctions in the pathobiology of periodontitis. Among the 24 studies included in this review, three recent studies [51–53] applied the new classification, categorizing periodontitis by stages and grades [19].

Considering the Armitage [54] classification, the included studies in this review showed that the most frequently reported DP in patients with chronic periodontitis was the ulnar loop [18, 20, 21, 36, 38, 39, 42, 43, 49, 51]. Vaidya et al. [21] observed that although the ulnar loop was more frequent in participants with chronic periodontitis, the prevalence of whorls was 21.3% in the periodontally healthy group compared to 41.1% in the chronic periodontitis group. This finding was corroborated by Elavarasu et al. [39], who reported a 20% higher occurrence of whorl patterns in the chronic periodontitis group compared to healthy participants. However, some studies reported divergent results, showing a higher prevalence of whorl patterns among patients with chronic periodontitis [35, 40, 44–48, 52].

Regarding aggressive periodontitis, Atasu et al. [36] found a prevalence of 52.8% of ulnar loop patterns, followed by whorls (38.9%) in patients with juvenile periodontitis (the term used in the 1989 classification; Armitage [54]). Similarly, Devishree and Gujjari [33] reported a 57% prevalence of ulnar loops and 30% of whorls in participants with aggressive periodontitis. In a recent study [50], the authors found similar results, with a higher prevalence of ulnar loops in patients diagnosed with aggressive periodontitis compared to other groups and a concomitant increase in whorl patterns. In another study, Grace and Sankari [45] also observed an increase in loops in aggressive periodontitis compared to healthy individuals, although the difference was not statistically significant (*p* = 0.897). Shyamala et al. [37] partially corroborated these findings, reporting higher occurrence of double loop whorls on the thumbs (60%) and single loop whorls on the left ring fingers (60%) in the aggressive periodontitis group but without the presence of ulnar loops.

These results should be interpreted with caution; although, the ulnar loop pattern was more prevalent in both individuals with chronic and aggressive periodontitis in the included studies, ulnar loops also represent the most prevalent fingerprint pattern in the general population, as demonstrated in studies that compared the DPs of different populations and between genders, which showed the prevalence of ulnar loops followed by whorls [55], or evaluated dermatoglyphics associated with other health conditions [10, 11, 16].

Periodontitis is a multifactorial disease, whose main etiological agent is a dysbiotic biofilm, modulated by systemic, immunological, and environmental determinants. Genetic alterations may also play a relevant role in the etiopathogenesis of periodontitis [18]. Genetic predisposition can result in an inadequate immune response to bacterial infection, and consequently, the individual becomes more susceptible to inflammation, which, combined with an excessively periodontopathogenic biofilm, will intensify the inflammatory response, leading to the destruction of periodontal tissues [56]. It is suggested that shared genetic factors may affect both the formation of DPs and the biological mechanisms related to predisposition to periodontitis [45]. However, this hypothesis lacks studies with molecular or genetic evidence to support it.

In this review, there was a significant geographic concentration of the included studies in a specific country (91.7% conducted in India), which may limit the generalizability of the results to other populations, especially given the influence of genetic, ethnic, socioeconomic, and environmental factors on both dermatoglyphics and periodontitis. The influence of genetic and ethnic variability on DPs is still poorly explored. Zhang et al. [57], when evaluating 56 ethnic groups in China, observed heterogeneity in the distribution of DPs among different groups within the same national population, in addition to differences associated with historical processes of migration and miscegenation. Complementarily, Li et al. [58], through a genome‐wide association study (GWAS), demonstrated that genes involved in the embryonic development of limbs and skin contribute to the formation of these patterns. It was also found that although the underlying genetic architecture is shared among populations, differences in allele frequencies and genetic ancestry result in ethnic and geographic variations in DPs. These findings may indicate that dermatoglyphics is influenced by individual and population genetic variability. Small random variations inherent in the developmental process amplify minimal differences between individuals, contributing to the uniqueness of fingerprints [4], demonstrating that dermatoglyphic variability has a complex biological basis, and these variations can influence the reproducibility and external validity of the results.

In addition, the marked methodological heterogeneity across studies represents an important limitation. Variability was observed in the diagnostic criteria for periodontitis—with different clinical indices applied and, in some cases, no description of diagnostic parameters. These differences can lead to the inclusion of individuals with distinct clinical characteristics under the same periodontal diagnosis, compromising the comparability between studies and consequently reducing the robustness of the evidence. Also in the fingerprint collection methods, which ranged from manual techniques (ink and paper) [18, 20, 21, 33–39, 42–44, 48, 49, 51, 52] to digital scanners [40, 46, 47, 50, 53] and direct visual inspection (magnifying lens) [45]. Such methodological diversity may affect comparability across studies and reduce the robustness of the conclusions.

Another aspect to highlight was the inconsistent reporting of key sociodemographic data, such as age and sex distribution, which restricts the analysis of potential biases related to these variables. Age is a critical determinant in the progression and clinical presentation of periodontitis. Studies show that individuals aged 65 years or older have a higher prevalence and severity of periodontitis. Therefore, age should be considered a modifying and confounding factor in the interpretation of the results found in the studies [59–61].

The risk of bias assessment revealed critical weaknesses, particularly in the domains of confounding factors and appropriateness of statistical analysis. Several studies used inadequate statistical analyses, using tests designed for ordinal or continuous variables, such as ANOVA [20, 51], Mann–Whitney [42], paired *t*‐test [38], and independent *t*‐test [21], even though DPs are categorical variables. The inappropriate use of statistical tests for categorical variables can lead to results reported as statistically significant when they are actually oscillations in the sample or uneven frequency distributions, leading to a false‐positive result. For these variables, the most appropriate tests are the chi‐square test or, when any category has an expected frequency *p*  < 0, 05, Fisher’s exact test, failed to describe the statistical tests performed [49, 50], or did not conduct statistical analyses at all [37, 39]. These shortcomings compromise the reliability of the findings and may reflect the absence of appropriate methodological and statistical design strategies.

This scoping review was conducted due to the exploratory nature and methodological heterogeneity of the literature on the use of dermatoglyphics as an auxiliary tool for periodontal disease detection. Preliminary searches revealed substantial variability across studies regarding study designs, periodontal diagnostic criteria, DPs, data collection and analysis methods, as well as investigated populations and outcomes. Given this diversity, a scoping review was considered the most appropriate approach to comprehensively map the available evidence, characterize existing methodological approaches, and identify knowledge gaps that may inform future research and more focused systematic reviews.

In clinical practice, identifying individuals at higher risk of developing periodontitis may support the implementation of more targeted and effective preventive strategies. DPs have several potentially advantageous features, including uniqueness, stability over time, ease of assessment, noninvasiveness, and low cost [8, 9], which may favor their clinical applicability. However, established clinical parameters, molecular biomarkers, genetic markers, and diagnostic imaging currently provide more robust approaches for assessing the susceptibility to and progression of periodontal diseases. In this context, dermatoglyphics may be considered a complementary phenotypic trait rather than a standalone diagnostic or predictive marker [53].

None of the analyzed studies presented diagnostic accuracy indices, such as sensitivity, specificity, or predictive values of DPs, compared to clinical periodontal diagnosis. Additionally, the methodological weaknesses of the studies, such as the variability in methods for evaluating DPs and heterogeneous periodontal diagnostic criteria and the lack of characteristics of the evaluated samples, directly impact the certainty of the evidence. Taken together, these findings highlight the need for future studies with greater methodological rigor, standardized diagnostic criteria, and samples that are more representative in order to strengthen both the internal and external validity of the evidence in this field.

## 5. Conclusions

Based on the included studies, a predominance of the ulnar loop pattern was observed among individuals with periodontitis, in both chronic and aggressive forms; this finding should be interpreted cautiously as ulnar loops are also the most prevalent fingerprint pattern in the general population. Some studies also reported a trend toward an increased frequency of whorl patterns, suggesting that this marker may reflect variations in development associated with susceptibility to periodontitis.

Although the results may suggest an association between certain DPs and the presence of periodontitis, it is still not possible to state that dermatoglyphics can be applied as an auxiliary diagnostic tool in the periodontal clinical practice. Future multicentric studies, including diverse populations, rigorous methodological design, and application of updated diagnostic criteria, are required to assess the true potential of dermatoglyphics as an auxiliary diagnostic method for periodontitis.

### 5.1. Implications for Clinical Practice

At present, dermatoglyphics should not be used as a standalone diagnostic method for periodontitis. While it may offer a low‐cost complementary approach in the future, current evidence does not support its routine clinical use.

### 5.2. Implications for Research

Future studies should adopt standardized periodontal diagnostic criteria, robust methodological designs, and adequate control of confounding factors, preferably through multicenter collaborations, to clarify the diagnostic value of dermatoglyphics in periodontitis.

## Author Contributions

Conceptualization, project administration: Virgílio Moreira Roriz. Methodology: Túlio Eduardo Nogueira. Investigation, formal analysis: Maria Teresa Andrade do Amaral, Maria Luiza Lima Santana, and Sara Maria do Amaral Melo. Data curation: Maria Teresa Andrade do Amaral and Sara Maria do Amaral Melo. Visualization, writing – original draft: Sara Maria do Amaral Melo. Writing – review and editing, supervision: Túlio Eduardo Nogueira and Virgílio Moreira Roriz.

## Funding

This study did not receive any specific funding.

## Disclosure

All authors have read and approved the final version of the manuscript. Virgílio Moreira Roriz had full access to all of the data in this study and takes complete responsibility for the integrity of the data and the accuracy of the data analysis.

## Ethics Statement

The authors have nothing to report.

## Conflicts of Interest

The authors declare no conflicts of interest.

## Supporting Information

Additional supporting information can be found online in the Supporting Information section.

## Supporting information


**Supporting Information 1** Supporting file 1: PRISMA Extension for Scoping Reviews (PRISMA‐ScR) checklist. This file contains the completed PRISMA‐ScR checklist detailing where each reporting item is addressed in the manuscript.

**Supporting Information 2** Supporting file 2:. Detailed search strategies for all databases. This file presents the full electronic search strategy for all databases.

## Data Availability

The data that support the findings of this study are available from the corresponding author upon reasonable request.
