# Supplementary material for: Is There Evidence Supporting the use of Dermatoglyphics as an Auxiliary Diagnostic Method for Periodontitis? A Scoping Review
Source: Int J Dent. 2026 Oct 8;2026:5546489. doi: 10.1155/ijod/5546489 (PMC13647206; doi:10.1155/ijod/5546489)
Supplement: Supplementary file 1 [file IJOD-2026-5546489-s001.docx]

**Supplementary File 1 –** Search strategy for each database

| Database | Search strategy | Records identified |
| --- | --- | --- |
| Medline / PubMed | ("Dermatoglyphics"[MeSH Terms] OR "dermatoglyph*"[Text Word] OR "fingerprint*"[Title/Abstract] OR "finger print*"[Title/Abstract] OR "skin ridge pattern*"[Title/Abstract] OR "dactyloscopy"[Title/Abstract] OR "ridge pattern"[Title/Abstract] OR "hand print"[Title/Abstract] OR "hand pattern"[Title/Abstract] OR "handprint"[Title/Abstract] OR "palm print"[Title/Abstract] OR "palm pattern"[Title/Abstract] OR "palmprint"[Title/Abstract]) AND ("Periodontitis"[MeSH Terms] OR "periodontiti*"[Title/Abstract] OR "Periodontitides"[Title/Abstract] OR "Periodontal Diseases"[MeSH Terms] OR "periodontal disease*"[Title/Abstract] OR "Parodontosis"[Title/Abstract] OR "Parodontoses"[Title/Abstract] OR "Pyorrhea Alveolaris"[Title/Abstract]) | 103 |
| Embase | ('dermatoglyphics'/exp OR 'dermatoglyph*':ti,ab,kw,de,dn,df,mn,tn OR 'fingerprint*':ti,ab,kw OR 'finger print*':ti,ab,kw OR 'skin ridge pattern*':ti,ab,kw OR 'dactyloscopy':ti,ab,kw OR 'ridge pattern':ti,ab,kw OR 'hand print':ti,ab,kw OR 'hand pattern':ti,ab,kw OR 'handprint':ti,ab,kw OR 'palm print':ti,ab,kw OR 'palm pattern':ti,ab,kw OR 'palmprint':ti,ab,kw) AND ('periodontitis'/exp OR 'periodontiti*':ti,ab,kw OR 'periodontitides':ti,ab,kw OR 'periodontal disease'/exp OR 'periodontal disease*':ti,ab,kw OR 'parodontosis':ti,ab,kw OR 'parodontoses':ti,ab,kw OR 'pyorrhea alveolaris':ti,ab,kw) | 126 |
| Scopus | TITLE-ABS-KEY(dermatoglyph* OR Fingerprint* OR "finger print*" OR "skin ridge pattern*" OR dactyloscopy OR "ridge pattern" OR "hand print" OR "hand pattern" OR handprint OR "palm print" OR "palm pattern" OR palmprint) AND TITLE-ABS-KEY(Periodontiti* OR "Periodontal Disease*" OR Parodontosis OR Parodontoses OR "Pyorrhea Alveolaris") | 250 |
| Web of Science | TS=(dermatoglyph* OR Fingerprint* OR "finger print*" OR "skin ridge pattern*" OR dactyloscopy OR "ridge pattern" OR "hand print" OR "hand pattern" OR handprint OR "palm print" OR "palm pattern" OR palmprint) AND TS=(Periodontiti* OR "Periodontal Disease*" OR Parodontosis OR Parodontoses OR "Pyorrhea Alveolaris") | 112 |
| Cochrane Library | ((dermatoglyph* OR Fingerprint* OR (finger NEXT print*) OR ("skin ridge" NEXT pattern*) OR dactyloscopy OR "ridge pattern" OR "hand print" OR "hand pattern" OR handprint OR "palm print" OR "palm pattern" OR palmprint)) AND ((Periodontiti* OR (Periodontal NEXT Disease*) OR Parodontosis OR Parodontoses OR "Pyorrhea Alveolaris")) | 05 |
| LILACS | (dermatoglyphic OR dermatoglyphics OR dermatoglyph OR dermatoglyphes OR dermatoglyphical OR dermatoglyphs OR fingerprint OR fingerprints OR "finger print" OR "finger prints" OR fingerprinting OR "skin ridge pattern" OR "skin ridge patterns" OR dactyloscopy OR "ridge pattern" OR "hand print" OR "hand pattern" OR handprint OR "palm print" OR "palm pattern" OR palmprint OR dermatoglifia OR dermatoglifos OR "Impressões Digitais" OR "Impresiones Digitales" OR "huellas de los dedos" OR "impressão digital" OR "huella dactilar" OR "huellas dactilares" OR "huella digital") AND (periodontitis OR periodontitides OR "Periodontal Diseases" OR "Periodontal Disease" OR parodontosis OR parodontoses OR "Pyorrhea Alveolaris" OR periodontite OR "Doenças Periodontais" OR "Doença Periodontal" OR paradontose OR parodontose OR "Piorreia Alveolar" OR "Enfermedades Periodontales" OR paradontosis OR "Piorrea Alveolar") AND db:("LILACS") AND instance:"regional" | 03 |
| ProQuest Dissertations & Theses Citation Index | TS=(dermatoglyph* OR Fingerprint* OR "finger print*" OR "skin ridge pattern*" OR dactyloscopy OR "ridge pattern" OR "hand print" OR "hand pattern" OR handprint OR "palm print" OR "palm pattern" OR palmprint) AND TS=(Periodontiti* OR "Periodontal Disease*" OR Parodontosis OR Parodontoses OR "Pyorrhea Alveolaris") | 11 |
| LIVIVO | (dermatoglyphic OR Dermatoglyphics OR dermatoglyph OR dermatoglyphes OR dermatoglyphical OR dermatoglyphs OR Fingerprint OR Fingerprints OR "finger print" OR "finger prints" OR Fingerprinting OR "skin ridge pattern" OR "skin ridge patterns" OR dactyloscopy OR "ridge pattern" OR "hand print" OR "hand pattern" OR handprint OR "palm print" OR "palm pattern" OR palmprint) AND (Periodontitis OR Periodontitides OR "Periodontal Diseases" OR "Periodontal Disease" OR Parodontosis OR Parodontoses OR "Pyorrhea Alveolaris") | 128 |
| Google Scholar | (Periodontitis OR Periodontitides OR "Periodontal Diseases" OR "Periodontal Disease" OR Parodontosis OR Parodontoses OR "Pyorrhea Alveolaris" OR Periodontite OR "Doenças Periodontais" OR "Doença Periodontal" OR Paradontose OR Parodontose OR "Piorreia Alveolar" OR "Enfermedades Periodontales" OR Paradontosis OR "Piorrea Alveolar") AND (dermatoglyphic OR Dermatoglyphics OR dermatoglyph OR dermatoglyphes OR dermatoglyphical OR dermatoglyphs OR Fingerprint OR Fingerprints OR "finger print" OR "finger prints" OR Fingerprinting OR "skin ridge pattern" OR "skin ridge patterns" OR dactyloscopy OR "ridge pattern" OR "hand print" OR "hand pattern" OR handprint OR "palm print" OR "palm pattern" OR palmprint OR Dermatoglifia OR Dermatoglifos OR "Impressões Digitais" OR "Impresiones Digitales" OR "huellas de los dedos" OR "impressão digital" OR "huella dactilar" OR "huellas dactilares" OR "huella digital") | 100 |
